# Danish general practitioners’ management of patients with COPD: a nationwide survey

**DOI:** 10.1080/02813432.2020.1842964

**Published:** 2020-11-09

**Authors:** Katrine Rutkær Molin, Jens Søndergaard, Peter Lange, Ingrid Egerod, Henning Langberg, Jesper Lykkegaard

**Affiliations:** a Section of Social Medicine, Department of Public Health, University of Copenhagen, Copenhagen, Denmark; b Research Unit of General Practice, Department of Public Health, University of Southern Denmark, Odense, Denmark; c Section of Epidemiology, Department of Public Health, University of Copenhagen, Copenhagen, Denmark; d Department of Medical, Herlev and Gentofte Hospital, Herlev, Denmark; e Clinical Nursing, Department of Intensive Care, Rigshospitalet, University of Copenhagen, Copenhagen, Denmark; f Section of Health Services Research, Department of Public Health, University of Copenhagen, Copenhagen, Denmark

**Keywords:** Chronic obstructive pulmonary disease, general practitioners, management, follow-up, family practice, educational support

## Abstract

**Background:**

In Denmark, general practitioners (GPs) have the main responsibility for chronic obstructive pulmonary disease (COPD) management. Internationally, COPD appears to be significantly under-treated, which could be explained by ‘therapeutic nihilism’ or lack of knowledge.

**Aim:**

To investigate: (1) To what extent COPD management provided by GPs includes the core elements of pharmacological treatment, smoking cessation and physical activity, and (2) To what extent GPs need educational support and consulting with a specialist in pulmonary medicine.

**Design:**

A national cross-sectional web-based survey conducted in April–June 2019. The survey included items on COPD management and educational support needs.

**Setting:**

Danish general practice.

**Subjects:**

A population of approximately 3400 GPs (all GPs in Denmark).

**Results:**

We received response from 470 GPs (14% response rate). Overall, the respondents reported that they offered COPD management including all relevant treatment elements. Smoking cessation was supported in 58% and physical activity was supported in 23% of the respondents. Future consultations on smoking cessation were planned by 35% and physical activity by 15% respondents. GPs responded to ‘needing educational support in COPD management’ to a ‘high degree’ in 8% and to ‘some degree’ in 43%.

**Conclusion:**

The survey suggested that COPD maintenance support provided by GPs seemed to be inadequate regarding smoking cessation and physical activity. Moreover, some GPs expressed a need for educational support in COPD management. More research is needed to understand the potential barriers to evidence-based delivery of COPD-management.

**Key points**
- In Denmark, general practitioners (GPs) have the main responsibility for the management of chronic obstructive pulmonary disease (COPD).
- The present study shows that non-pharmacological interventions such as supporting smoking cessation and particularly promoting physical activity received less attention than pharmacological treatment.
- The study suggests a need for educational support of the GPs in COPD management.

## Introduction

In Denmark, general practitioners (GPs) have the main responsibility for chronic obstructive pulmonary disease (COPD) management. This responsibility has been strengthened in the present agreement between the Danish Regions and the General Practitioners Union (OK18) [1], with the new structure limiting hospital and pulmonary specialist referrals. General practice in Denmark is the key element of primary health care, where the GP is the gatekeeper and first-line provider to the secondary healthcare system in the sense that in- and outpatient hospital treatment including most office-based specialists require a referral from a GP [2,3]. Therefore, the GP is responsible for minimizing unnecessary referrals. Thus, during the past two years, many patients with stable COPD have been transferred from hospital-based outpatient clinics to general practice [1]. The GP is responsible for the provision of COPD management that includes three core elements: Pharmacological treatment, smoking cessation, and physical activity [3–7]. The GP may offer counseling and motivation or may refer the patient to a community-based COPD rehabilitation program [5–7]. The GP is responsible for referring patients to a rehabilitation program while the municipalities have the responsibility for the content and execution of the program. As the patients’ gatekeeper and facilitator, once the program is completed, the GP is responsible for ensuring the best possible maintenance of the lifestyle changes achieved [5–7]. Finally, the present agreement (OK18) gives the GPs better options for consulting with a specialist in pulmonary medicine [1].

Internationally, a substantial under-treatment of patients with COPD has been reported [8–10]. One explanation could be ‘therapeutic nihilism’ where the GP regards COPD treatment as futile compared to treatment of other chronic diseases [8,9]. Another possibility is that GPs’ COPD knowledge could be insufficient [11,12]. Nevertheless, the low priority of COPD management can be attributed to both patients and healthcare professionals [12–16]. The patients seem to visit the GP too rarely [12,13,16] and the efforts made by the GPs seem inadequate [13–16].

With the new allocation of COPD responsibilities to GPs and previous research in mind, the aim of this study was to investigate: (1) To what extent COPD management provided by GPs includes the core elements of pharmacological treatment, smoking cessation and physical activity, and (2) To what extent GPs need educational support and consulting with a specialist in pulmonary medicine.

## Methods

### Study design

We conducted a national cross-sectional web-based survey assessing GPs’ COPD management in Danish general practices. Data were generated in April–June 2019 as part of a larger study on perception and management of COPD [13,14]. On 24 April, a letter was sent by regular mail to the existing 1766 general practices, inviting approximately 3400 GPs to participate in this study. After four weeks, the 21 May, we sent a reminder, and the survey closed on 7 June. We offered the GPs EUR 19 to compensate for the time to answer the questionnaire, which was estimated at 10 minutes.

The questionnaire was developed based on the scientific literature, including our own studies and on clinical experience. Before sending out the questionnaire, it was piloted in a two-step procedure: (1) Questionnaire completed and commented by 11 GPs with expert research knowledge in the field, and (2) a revised questionnaire, based on the pilot, completed and commented by six random GPs. In the second phase, we also conducted two telephone interviews after the GPs had completed the questionnaire. All approaches were beneficial for the development of the final version of the questionnaire.

We used SurveyXact constructing a web-based survey; the survey was available on the website www.KOL2019.dk. Information regarding the website as well as a recommendation letter by the Danish Committee of Multipractice Studies in General Practice for GPs to participate in this study was included in the invitation letter.

### Measures included in the questionnaire

GPs were asked questions related to their demographic characteristics: Sex, age, years of experience, type of practice, and municipality code for general practice. Further, questions concerned the GPs perception of their COPD management and support needs, respectively, Table 1.

**Table 1.** Items in the questionnaire used to assess GPs COPD management and GPs support needs.

Items covered in each topic	Response categories
COPD management	
1. ‘What proportion of your patients with diagnosed COPD have been advised about the treatment elements being pharmacological treatment, physical activity, smoking cessation, and referral to COPD rehabilitation?’	(Likert response scale) Nearly all, majority, about half, a few, almost none, don’t know
2. ‘To what extent are the following elements included in your conversation about physical activity with patients with COPD?’ (Explain benefits, define goals with the patient, plan new consultations, refer to COPD rehabilitation)	(Likert response scale) Very high degree, high degree, some degree, low degree, very low degree, not at all, don’t know
3. ‘To what extent are the following elements included in your conversation about smoking cessation with patients with COPD?’ (Explain benefits, define goals with the patient, plan new consultations, refer to COPD rehabilitation)	(Likert response scale) Very high degree, high degree, some degree, low degree, very low degree, not at all, don’t know
4. ‘From your point of view, to what extent do the patients benefit from a COPD rehabilitation program?’	(Likert response scale) Very high degree, high degree, some degree, low degree, very low degree, not at all, don’t know
Support needs	
5. ‘In the last year, have you had the need to consult with a specialist in pulmonary medicine regarding the treatment of one or more of your patients with COPD (with the exclusion of consultations about a potential referral to outpatient or acute hospitalization)?’	(Binary response) Yes, no or don’t know
6. ‘Was it easy for you to get access to consult with a specialist in pulmonary medicine?’	(Likert response scale) Very high degree, high degree, some degree, low degree, very low degree, not at all, don’t know
7. ‘Do you find that you need more consultations with a specialist in pulmonary medicine than what is currently available to you?’	(Likert response scale) Very high degree, high degree, some degree, low degree, very low degree, not at all, don’t know
8. ‘Do you find that you need educational support in COPD management?’	(Likert response scale) Very high degree, high degree, some degree, low degree, very low degree, not at all, don’t know
Demographic characteristics	
9. Sex?	Female, male
10. Age?	Select the correct number
11. For how many years have you been a GP?	Select the correct number
12. Which of the following terms fits best your practice?	Single-handed practice, cooperation practice, partnership practice
13. In which municipality is your practice located?	Select one of the 98 municipalities in Denmark

COPD: Chronic obstructive pulmonary disease; GP: General practitioner.

#### Items on COPD management

The GPs were asked about how many of their patients with COPD are given advice about the main treatment elements including pharmacological treatment, physical activity, smoking cessation, and referral to COPD rehabilitation, Table 1 (item 1). Further, the GPs were asked to what extent different elements were included in their dialog about physical activity and smoking cessation, Table 1 (item 2–3). Finally, GPs were asked to what extent they believed that the patients benefit from a COPD rehabilitation program, Table 1 (item 4).

#### Items on GPs support needs

The GPs were asked if they, within the last year, had needed to consult with a specialist in pulmonary medicine regarding the treatment of one or more of their patients with COPD, Table 1 (item 5). Depending on their answer to this question, GPs were asked whether they experienced the access to the specialist in pulmonary medicine as easy, Table 1 (item 6). Further, they were asked whether they needed to consult with a specialist in pulmonary medicine to a higher extent than what was currently available, Table 1 (item 7). Finally, the GPs were asked if they believed that they needed educational support in COPD management, Table 1 (item 8).

### Statistical analysis

We performed descriptive analysis to describe distribution and statistical variation. The demographic characteristics of GPs were used as explanatory variables. Many answers of the items related to COPD management and support needs were indicated on a six-point and seven-point Likert response scale, respectively, Table 1. In order to compare GPs, the values on the Likert response scale were dichotomized into high (‘very high or high degree’ and ‘almost all or majority’) or low degree (the rest of the categories). In the analysis, the answers in the category ‘don’t know’ was not included. We examined the responses using multiple regression models (models of linear probability). For all statistical analysis, we assessed a nominal two-sided five percent significance level.

### Ethics

The study complied with the newest version of the Declaration of Helsinki [17]. Respondent GPs gave informed written consent. The legal department at University College Copenhagen (case ID number 18-206) provided approval for this study.

## Results

### Demographic characteristics

In total, 527 GPs participated in the study of which 470 GPs completed the survey, yielding a response rate of 14% [18]. The gender distribution was 51/49 pct. (female/male) and mean age 51 years (range 32-75), Table 2. The distribution of respondents in the five regions of Denmark were representative of the background population of all Danish GPs [18]. Years of general practice experience was mean 13 years (range 1–43). Type of practice distribution was single-handed practice 21% (a practice operated by a single physician [19]), cooperation practice 14% (a practice operated by more than one physician sharing equipment but with separate economy [19]), and partnership practice 64% (a practice operated by more than one physician sharing patients, staff and economy [19]).

**Table 2.** Demographic characteristics.

	Respondents (*n* = 470)	GP Background population (*N* = 3365)
Sex		
Male	49%	51%
Female	51%	49%
Age		
30-39	43 (9%)	247 (7%)
40-49	195 (41%)	1315 (39%)
50-59	136 (29%)	964 (29%)
60-69	91 (19%)	776 (23%)
70-79	5 (1%)	63 (2%)
Years of experience		
0-9	200 (43%)	
10-19	149 (32%)	
20-29	98 (21%)	
30-39	21 (4%)	
40-49	2 (0%)	
Type of practice		
Single-handed practice	100 (21%)	≈ 40%
Cooperation practice	67 (14%)	≈ 20%
Partnership practice	303 (64%)	≈ 40%
The five regions of Denmark		
Central Denmark Region	135 (29%)	819 (23%)
North Denmark Region	41 (9%)	337 (10%)
Region of Southern Denmark	117 (25%)	786 (22%)
Capital Region of Denmark	123 (26%)	1062 (30%)
Region Zealand	54 (11%)	493 (14%)

### COPD treatment elements

All respondents reported that they talked to their patients about pharmacological treatment (98%) and that they discussed the importance of smoking cessation (100%). Further, 96% reported that they explained why physical activity was important. In this connection, 70% told their patients that a COPD rehabilitation program was available to them, and 62% reported that they believed to a high or very high degree that the patients benefit from such a program. In the present study, we were unable to assess the degree of patient involvement in these exchanges, but have described the patient perspective elsewhere [14].

**Figure 1.**
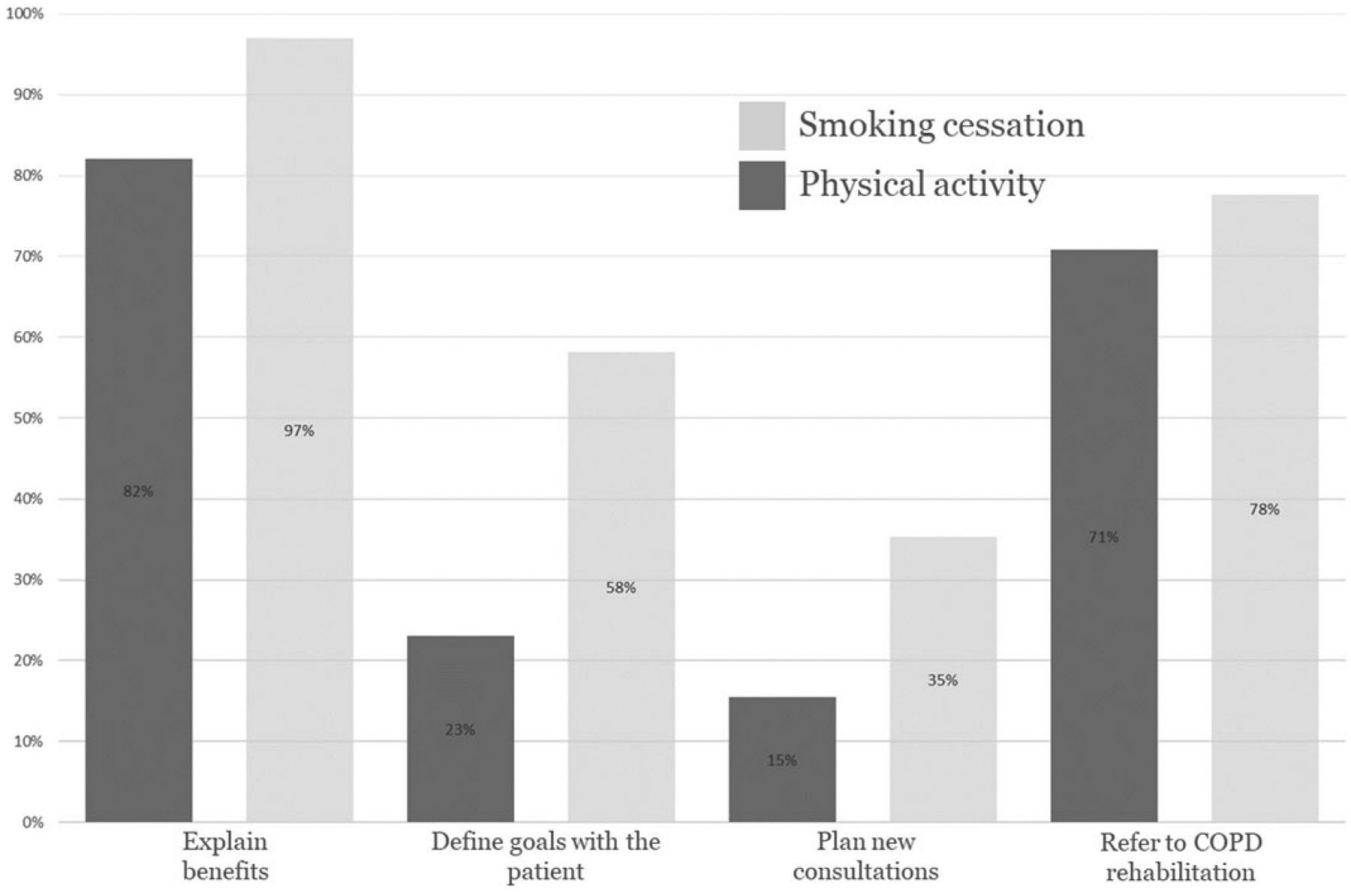
The engagement profile concerning smoking cessation and physical activity.

Regarding physical activity, 23% of the respondents defined specific targets for physical activity in collaboration with the patient. Further, 15% planned future consultations to motivate and support the patient to be physically active. Multiple regression gives some indication (although not high estimates) that it is more likely that GPs with more experience suggest future consultations (*p* < 0.05), Table 3. By contrast, 58% of the respondents were more likely to define specific targets for smoking cessation and 35% planned future consultations (Figure 1).

**Table 3.** Planning future consultations for physical activity/GPs support needs (Multiple regression analysis).

	Plan consultation for physical activity	Support needs
Female	−0.061* (0.035)	0.195*** (0.048)
Single-handed practice	0.017 *(0.043)*	−0.077 *(0.060)*
Years’ Experience	0.004** (0.002)	−0.005* (0.003)
Central Denmark Region	0.040 *(0.046)*	0.022 *(0.064)*
North Denmark Region	0.037 *(0.066)*	−0.184** (0.092)
Region of Southern Denmark	−0.009 *(0.047)*	−0.082 *(0.065)*
Region Zealand	0.024 *(0.059)*	0.017 *(0.082)*
Define goals for physical activity		0.043 *(0.030)*
Plan consultation for physical activity		0.071*** (0.027)
Define goals for smoking cessation		−0.023 *(0.030)*
Plan consultation for smoking cessation		−0.025 *(0.027)*
Constant	0.109** (0.049)	0.322** (0.133)
Observations	466	434
R2	0.026	0.103
Adjusted R2	0.011	0.080
Residual Std. Error	0.360 (d*f* = 458)	0.480 (d*f* = 422)
F Statistic	1.757* (d*f* = 7;458)	4.422* (d*f* = 11;422)

Note: *p < 0.1;**p < 0.05;***p < 0.01.

All the independent variables (besides ‘Years’ Experience’) are dummy variables in the regression analysis.

### GPs support needs

**Figure 2.**
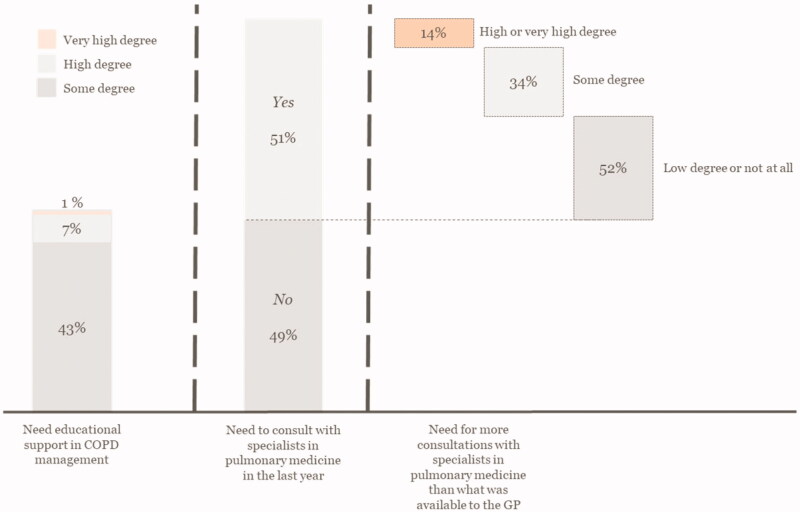
GPs support needs.

The GPs might not have been sufficiently prepared for the greater responsibility allotted to them in COPD management. The respondents reported that they, to a high (8%) or some (43%) degree, needed educational support in COPD management. About half of the respondents (51%) had consulted with a specialist in pulmonary medicine within the past year, and 14% needed more support than offered (Figure 2). Multiple regression suggested some geographical variations, as respondents in the North Denmark Region reported fewer support needs than respondents in the Capital Region of Denmark (*p* < 0.05). Interestingly, GPs recommending additional physical activity consultations, had a greater need to consult with specialists (*p* < 0.05), Table 3. About half of the respondents (54%) had easy access to specialists in pulmonary medicine, while 14% did not.

## Discussion

### Statement of principal findings

Most GPs in the present study reported that they advised the patients on the importance of smoking cessation, physical activity and pharmacological treatment. Many GPs talked to the patients about the possibility of referral to a COPD rehabilitation program, and reported that they believed that patients benefit from such a program. However, non-pharmacological interventions such as supporting smoking cessation and, particularly, physical activity received less attention than pharmacological treatment. About half of the respondents reported that they, at least to some degree, needed educational support in COPD management.

### Findings in relation to other studies

In the present study, GPs reported that their management of COPD included all relevant treatment elements. Concerning smoking cessation and physical activity, many respondents, however, did not go the step further to plan future consultations in collaboration with the patient. Also, they put more effort into supporting smoking cessation than physical activity. Thus, the difficult task of life-style change was up to the patient, which could be difficult to obtain in this population. Many patients with COPD are unsuccessful in smoking cessation [20], or lack motivation [21]. Studies have shown that the physician’s attitude to physical activity might have an impact on the patient’s motivation to engage in physical activity [22,23]. For this reason, monitoring and motivational consultations are essential [20].

It has been shown that physicians lack knowledge on how to help the patients to stop smoking and are frustrated by the patients’ smoking habits [24]. Physicians’ shortcomings in providing non-pharmacological treatment might also be attributed to organizational factors such as lack of financial incentive [13,24] and time constraints in general practice [24,25]. In Denmark, general practice is a semi-private branch of the otherwise tax-payed healthcare system [2]. In hospitals, the physicians get monthly wages, whereas GPs are paid quarterly fee per listed patient with COPD. In addition to these motivational factors, a Danish study found that physicians in primary care had more difficulty motivating patients with COPD than patients with type-2 diabetes [26]. Finally, GPs may not consider smoking cessation and physical activity as their responsibility because it is taken care of by the community COPD rehabilitation program [13]. These factors are all detrimental to the preventive effort in patients with COPD.

In our study, about half of the GPs stated that they, at least to some degree, needed educational support in COPD management. Similar studies show that some GPs stray from the guideline recommendations [10,11,13,14,27,28]. Our findings suggested variation according to the location; GPs from a rural area reported less need to consult a specialist than city GPs. Other studies have described similar findings [29,30]. One explanation put forth is that rural GPs have a broader scope of practice [29,30].

### Strengths and weaknesses of the study

Our study was limited by the low response rate. This is to be expected in this type of broad survey with an unsolicited and time-consuming questionnaire. However, the fact that the respondents were representative of the Danish GP population at large (Table 2) increases the validity of our findings. The respondents in our study did not focus on setting future goals in collaboration with the patient. We lack contextual information describing the patient response to the GPs efforts. In addition, we are unable to determine the referral rate to COPD rehabilitation programs. Notwithstanding, a high referral rate cannot replace GPs active engagement in setting goals and planning new sessions with the patient. There may have been a selection bias due to potential differences in characteristics between the GPs who responded to the questionnaire and those who did not. For example, respondents may have been more likely to complete the questionnaire if the subject were of interest to them, if they were frustrated by not being able to offer adequate COPD management, or if they were concerned about the present agreement regarding the allocation of care in patients with COPD [1]. That said, the invitation letter did not refer to the agreement and was presented in a different context. Finally, other studies [9,10,12,13,26,27] support many of our findings, providing external validity.

## Conclusion

The survey suggested that COPD maintenance support provided by GPs seemed to be inadequate regarding smoking cessation and physical activity. Moreover, some GPs expressed a need for educational support in COPD management. More research is needed to understand the potential barriers to evidence-based delivery of COPD-management.

## References

[1] Praktiserende Laegers Organisation (PLO). Redegørelse for forhandlingsaftalen af 14. september 2017. mellem RLTN og PLO. [Agreement between the Danish regions and the general practitioners trade Union (OK18)]. Praktiserende Laegers Organisation; 2018. Available at: https://www.laeger.dk/sites/default/files/redegoerelseok18nyny.pdf. (accessed 30 April 2018). Danish.

[2] Pedersen KM, Andersen JS, Søndergaard J. General Practice and Primary Health Care in Denmark. J Am Board Fam Med. 2012;2325(Suppl 1):S34–S8. 22403249 10.3122/jabfm.2012.02.110216

[3] Tønnesen H, Bendix AF, Hendriksen C, et al. Laegens rolle i rehabilitering [The Doctor’s Role in Rehabilitation]. København: Den Almindelige Danske Laegeforening – Sundhedskomiteen, Laegeforeningens Forlag; 2006.

[4] Global Initiative for Chronic Obstructive Pulmonary Disease. Global strategy for the diagnosis, management, and prevention of chronic obstructive pulmonary disease - 2020 report. Available at: https://goldcopd.org/gold-reports/. (accessed 5 February 2020.

[5] Sundhedsstyrelsen. Anbefalinger for tvaersektorielle forløb for mennesker med KOL. [Recommendations for cross-sectoral courses for people with COPD]. Sundhedsstyrelsen 2017:1-77. Available at: www.sst.dk. (accessed 8 April 2020). Danish.

[6] Region Hovedstaden. Forløbsprogram for KOL [COPD program]. Region Hovedstaden. 2015;:1–45. Available at: https://www.regionh.dk/til-fagfolk/Sundhed/Tvaersektorielt-samarbejde/kronisk-sygdom/Forløbsprogrammer/Documents/RH_KOL_Program_rev_2015%2024032016.pdf (accessed 8 April 2020). Danish.

[7] Dansk Selskab for Almen Medicin. KOL i almen praksis [COPD in general practice]. Dansk Selskab for Almen Medicin; 2017. Available at: https://vejledninger.dsam.dk/kol/. (accessed 5 May 2020). Danish.

[8] Fromer L, Barnes T, Garvey C, et al. Innovations to achieve excellence in COPD diagnosis and treatment in primary care. Postgrad Med. 2010;122(5):150–164. 20861599 10.3810/pgm.2010.09.2212

[9] Radin A, Cote C. Primary care of the patient with chronic obstructive pulmonary disease-part 1: frontline prevention and early diagnosis. Am J Med. 2008;121(7 Suppl):S3–S12. 10.1016/j.amjmed.2008.04.002 18558105

[10] Pirina P, Martinetti M, Spada C, COPD-HF Study Group, et al. Prevalence and management of COPD and heart failure comorbidity in the general practitioner setting. Respir Med. 2017;131:1–5. 28947013 10.1016/j.rmed.2017.07.059

[11] Sandelowsky H, Natalishvili N, Krakau I, et al. COPD management by Swedish general practitioners - baseline results of the PRIMAIR study. Scand J Prim Health Care. 2018;36(1):5–13. 29334861 10.1080/02813432.2018.1426148 PMC5901441

[12] Hyde N, Casey D, Murphy K, et al. COPD in primary care settings in Ireland: Stories from usual care. Br J Community Nurs. 2013;18(6):275–282. 24046924 10.12968/bjcn.2013.18.6.275

[13] Molin KR, Egerod I, Valentiner LS, et al. General practitioners' perceptions of COPD treatment: thematic analysis of qualitative interviews. Int J Chron Obstruct Pulmon Dis. 2016;11:1929–1937. 27574417 10.2147/COPD.S108611 PMC4994802

[14] Molin KR, Langberg H, Lange P, et al. Disease self-management in patients with moderate COPD: a thematic analysis. Eur Clin Respir J. 2020;7(1):1762376. 33224451 10.1080/20018525.2020.1762376 PMC7655073

[15] Halding A-G, Heggdal K, Wahl A. Experiences of self-blame and stigmatisation for self-infliction among individuals living with COPD. Scand J Caring Sci. 2011;25(1):100–107. 20534028 10.1111/j.1471-6712.2010.00796.x

[16] Nielsen PB, Witzel S. Regular control at the general practitioner is positively correlated with patient satisfaction with chronic care management. Dan Med J. 2016;63(3):1–5. 26931190

[17] World Medical Association. World Medical Association Declaration of Helsinki. Ethical Principles for Medical Research Involving Human Subjects. World Medical Association; 2013. Available at: https://www.wma.net/policies-post/wma-declaration-of-helsinki-ethical-principles-for-medical-research-involving-human-subjects/. (accessed 31 September 2019). 10.1001/jama.2013.281053 24141714

[18] Teilmann M, Skipper M, Worsoe M, et al. Laegedaekning i hele Danmark. Rapport. [Doctor covering throughout Denmark. Report]. Regeringens Laegedaekningsudvalg; 2017. Available at: https://sum.dk/∼/media/Filer%20-%20Publikationer_i_pdf/2017/Laedaekningsudvalgets-rapport/Laegedaekning-rap-jan-2017.pdf. (accessed 5 December 2019). Danish.

[19] Praktiserende laegers organisation. PLO analyse - der skal vaere plads til forskellige typer af almen praksisklinikker. [PLO analysis - there must be room for different types of primary care practices]. PLO; 2019. Available at: https://www.laeger.dk/plo-analyse. (accessed 7 November 2019). Danish.

[20] Liang J, Abramson MJ, Zwar NA, et al. Diagnosing COPD and supporting smoking cessation in general practice: evidence-practice gaps. Med J Aust. 2018;208(1):29–34. 29320670 10.5694/mja17.00664

[21] Danielsen SE, Løchen M-L, Medbø A, et al. A new diagnosis of asthma or COPD is linked to smoking cessation - the Tromsø study. Int J Chron Obstruct Pulmon Dis. 2016;11:1453–1458. 27418818 10.2147/COPD.S108046 PMC4934533

[22] Lanhers C, Duclos M, Guttmann A, et al. General practitioners’ barriers to prescribe physical activity: the dark side of the cluster effects on the physical activity of their type 2 diabetes patients. PLoS ONE. 2015;10(10):e0140429. 26468874 10.1371/journal.pone.0140429 PMC4607360

[23] Van Sluijs EMF, van Poppel MNM, Twisk JWR, et al. Effect of a tailored physical activity intervention delivered in general practice settings: results of a randomized controlled trial. Am J Public Health. 2005;95(10):1825–1831. 16186461 10.2105/AJPH.2004.044537 PMC1449443

[24] van Eerd EAM, Risør MB, Spigt M, et al. Why do physicians lack engagement with smoking cessation treatment in their COPD patients? A multinational qualitative study. NPJ Prim Care Respir Med. 2017;23(1):1–6. 10.1038/s41533-017-0038-6 PMC5482893 28646217

[25] Sandelowsky H, Hylander I, Krakau I, et al. Time pressured deprioritization of COPD in primary care: a qualitative study. Scand J Prim Health Care. 2016;34(1):55–65. 26849465 10.3109/02813432.2015.1132892 PMC4911027

[26] Fredslund EK, Larsen AT, Klausen MB, et al. Almen praksis’ rolle i forhold til borgere med kroniske lidelser. En analyse af almen praksis med fokus på forebyggelse af sygehuskontakter hos KOL- og diabetespatienter. [The role of primary care practice in relation to citizens with chronic conditions. An analysis of primary care practice focusing on the prevention of hospital contacts in COPD and diabetes patients]. VIVE – Viden til Velfaerd Det Nationale Forsknings- og Analysecenter for Velfaerd; 2020. Available at: https://simb.dk/media/37817/almen-praksis-rolle-i-forhold-til-borgere-med-kroniske-lidelser.pdf. (accessed 29 March 2020). Danish.

[27] Reddel HK, Valenti L, Easton KL, et al. Assessment and management of asthma and chronic obstructive pulmonary disease in Australian general practice. Aust Fam Physician. 2017;46(6):413–419. 28609599

[28] Davis KJ, Landis SH, Oh Y-M, et al. Continuing to Confront COPD International Physician Survey: physician knowledge and application of COPD management guidelines in 12 countries. Int J Chron Obstruct Pulmon Dis. 2014;10:39–55. 25565799 10.2147/COPD.S70162 PMC4284025

[29] Pohontsch NJ, Hansen H, Schäfer I, et al. General practitioners' perception of being a doctor in urban vs. rural regions in Germany - A focus group study. Fam Pract. 2018;35(2):209–215. 29029048 10.1093/fampra/cmx083 PMC5892171

[30] Hanks H, Veitch PC, Harris MF. A rural/urban comparison of the roles of the general practitioner in colorectal cancer management. Aust J Rural Health. 2008;16(6):376–382. 19032211 10.1111/j.1440-1584.2008.01019.x

